# Environmental Enrichment Decreases Asphyxia-Induced Neurobehavioral Developmental Delay in Neonatal Rats

**DOI:** 10.3390/ijms141122258

**Published:** 2013-11-13

**Authors:** Peter Kiss, Gyongyver Vadasz, Blanka Kiss-Illes, Gabor Horvath, Andrea Tamas, Dora Reglodi, Miklos Koppan

**Affiliations:** 1 Department of Anatomy, PTE-MTA “Lendulet” PACAP Research Team, University of Pecs, Pecs 7624, Hungary; E-Mails: peter.kiss@aok.pte.hu (P.K.); vadaszgyongyi@gmail.com (G.V.); blanka.illes@gmail.com (B.K.-I.); gabor.horvathmd@gmail.com (G.H.); andreatamassz@gmail.com (A.T.); 2 Department of Obstetrics and Gynecology, University of Pecs, Pecs 7624, Hungary; E-Mail: mkoppan@gmail.com

**Keywords:** asphyxia, enriched environment, reflex development, motor coordination

## Abstract

Perinatal asphyxia during delivery produces long-term disability and represents a major problem in neonatal and pediatric care. Numerous neuroprotective approaches have been described to decrease the effects of perinatal asphyxia. Enriched environment is a popular strategy to counteract nervous system injuries. The aim of the present study was to investigate whether enriched environment is able to decrease the asphyxia-induced neurobehavioral developmental delay in neonatal rats. Asphyxia was induced in ready-to-deliver mothers by removing the pups by caesarian section after 15 min of asphyxia. Somatic and neurobehavioral development was tested daily and motor coordination weekly. Our results show that rats undergoing perinatal asphyxia had a marked developmental delay and worse performance in motor coordination tests. However, pups kept in enriched environment showed a decrease in the developmental delay observed in control asphyctic pups. Rats growing up in enriched environment did not show decrease in weight gain after the first week and the delay in reflex appearance was not as marked as in control rats. In addition, the development of motor coordination was not as strikingly delayed as in the control group. Short-term neurofunctional outcome are known to correlate with long-term deficits. Our results thus show that enriched environment could be a powerful strategy to decrease the deleterious developmental effects of perinatal asphyxia.

## 1. Introduction

Perinatal asphyxia during delivery produces long-term deficits and represents a major problem in neonatal and pediatric care [1–3]. It is based on a temporary interruption of oxygen availability that causes metabolic challenge, even when the distress does not lead to a fatal outcome [4]. Different clinical parameters have been used to both diagnose and predict the prognosis for perinatal asphyxia, and clinically, this type of brain injury is called hypoxic-ischemic encephalopathy (HIE). Sarnat and Sarnat proposed a staging system in 1976 that is useful in classifying the degree of encephalopathy. Different stages are commonly diagnosed using physical examination, which evaluates the level of consciousness, neuromuscular control, reflexes, pupils, heart rate, bronchial and salivary secretions, bowel motility, presence or absence of myoclonus or seizures, autonomic function and electroencephalography findings [5]. However, long-term neurologic injury cannot be predicted by these parameters after mild to moderate asphyxia [6].

The mechanisms of neuronal damage and cell death after perinatal asphyxia includes necrosis, apoptosis, and autophagia, mainly depending on the severity of the insult and the state of maturation of the cell [4,7–10]. Moreover, transient increase in excitatory amino acid levels has been found in the cerebrospinal fluid of human newborns and in several experimental models [11–14]. Also, extensive research has recently been focused on a potential link between the immune and neuronal systems, mainly in the context of pathogenesis, in which sustained or excessive inflammation has been associated with neurotoxicity and several neuropathologies [15–18]. It is widely accepted that early intervention and neuroprotection are necessary to improve the overall outcome, involving the inhibition of various potentially destructive molecular pathways, such as excitotoxicity, inflammation, oxidative stress and cell death. Moreover, therapies aiming to restore functionality of neurocircuitries by stimulation of neurotrophic endogenous properties of the neonatal brain are also of great importance.

There are several ways to mimic perinatal asphyxia or some of its features in animals. Some models use hypoxic exposure in 7-day-old rats, the developmental stage of which closely resembles that of the human newborn [19]. Other models use perinatal hypoxic exposure in pups removed from ready-to-deliver mothers or exposing the mother to hypoxia before birth [20–23]. These models mimic the pathophysiological processes at the time of delivery to a closer extent.

Short-term neurofunctional outcome has been shown to correlate with long-term functional deficits, which draws the attention to the predictive value and necessity of short-term evaluation [24]. Postnatal development is well-reflected in the maturation of neurological reflexes and motor coordination. In our earlier studies, we have described the neurobehavioral development in pups exposed to various injuries: excitotoxic injury, maternal deprivation and neonatal hypoxia [25–27]. We found that hypoxic injury causes the most severe neurobehavioral delay. As perinatal asphyxia is a severe hypoxic injury, we have also studied the effect of perinatal asphyxia on the developmental maturation. In a previous study we have shown that pups exposed to perinatal asphyxia have a marked delay in the neurobehavioral development [28]. Somatic development and the appearance of neurological reflexes were delayed by 1–4 days. The described delay was the most severe delay found among our models of perinatal injuries.

Neuroprotective strategies aim at counteracting the deleterious effects of brain injuries. Numerous neuroprotective approaches have been described to decrease the effects of perinatal asphyxia. Among others, beneficial effects of hypothermia [29], nicotinamide [30], preconditioning [31], erythropoietin [32], melatonin [33] and calcitriol [34] have been described. Enriched environment is a popular strategy used in several nervous system injuries. Following the first description of environmental enrichment by the influential neuroscientist Donald Hebb [35], data have accumulated showing that enriched environment not only enhances cognitive performance but it has protective effects in several types of brain injuries [36]. We have described that enriched environment protects neonatal rat retinas against glutamate-induced excitotoxic lesion and also adult retinas against ischemic lesion [37,38]. Our most recent study has provided evidence that enriched environment decreases the neurobehavioral delay caused by neonatal excitotoxic lesion [39]. The aim of our present study was to investigate whether early environmental enrichment is able to reverse the negative effects of perinatal asphyxia on neurobehavioral development.

## 2. Results and Discussion

### 2.1. Somatic Development

Both acute mortality and death during the observation period was high among the asphyctic pups in contrast to the control group, where mortality was much lower, in accordance with our previous results [28]. Only data from rats surviving during the whole observation period were included in the study (*n* = 21 in the asphyctic group and *n* = 25 in the control group).

**Figure 1:**
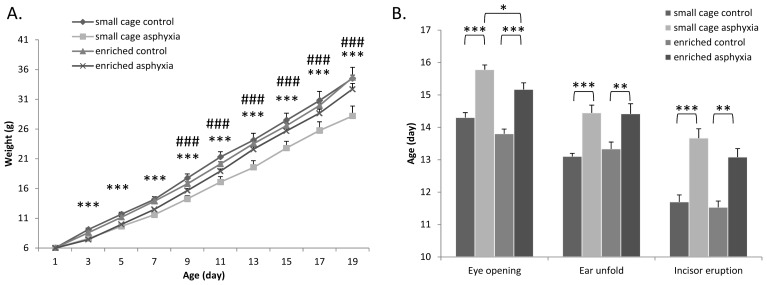
(**A**) Daily changes in body weight (*** *p* < 0.001 small cage control *vs.* small cage asphyctic group; ^###^*p* < 0.001 small cage asphyctic *vs.* enriched asphyctic group); and (**B**) Appearance of physical parameters (eye opening, ear unfold, incisor eruption; * *p* < 0.05, ** *p* < 0.01, *** *p* < 0.001).

Asphyctic pups had significantly less weight gain than control groups, especially during the second week of the observation period (Figure 1A). However, there was no difference in the body weight of asphyctic pups growing up in enriched environment after the first week. Enriched environment alone did not lead to increased weight gain in control animals. These data indicate that enriched environment can prevent the reduced weight gain in a neonatal asphyctic lesion.

### 2.2. Appearance of Physical Signs

There was no significant difference in the day of the appearance of eye opening, ear unfolding and incisor eruption between control pups kept in small cages or in environmental enrichment (Figure 1B). However, there was significant delay in rats undergoing perinatal asphyxia (Figure 1B). Delays of 1–2.5 days in asphyctic pups compared to the respective control groups could be observed. This delay in eye opening could be significantly decreased in pups under environmental enrichment. Although not at a significant level, a similar tendency was observed in case of incisor eruption.

### 2.3. Appearance of Reflexes

**Figure 2:**
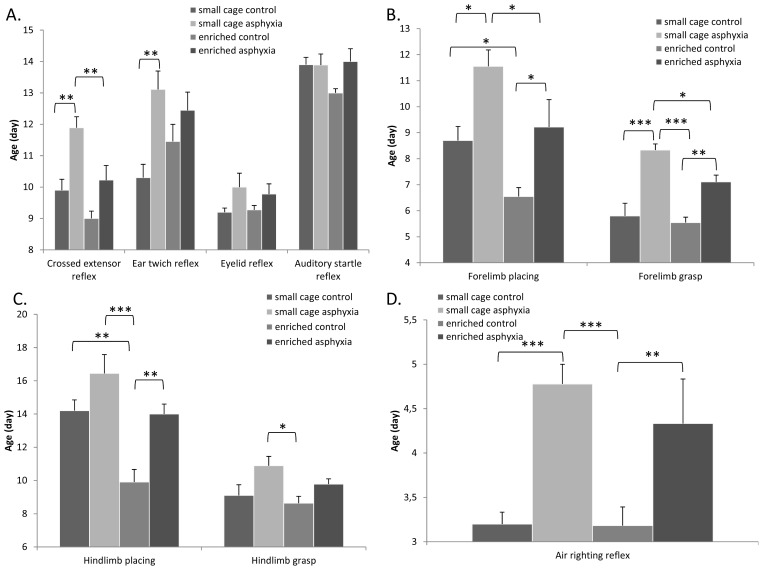
(**A**) Appearance of crossed extensor reflex, ear twitch-, eyelid reflex and auditory startle reflex; (**B**) Appearance of forelimb placing and grasp; (**C**) Appearance of hindlimb placing and grasp; and (**D**) Air righting reflex appearance (* *p* < 0.05, ** *p* < 0.01, *** *p* < 0.001).

According to our earlier observations [28], almost all neurological reflexes were delayed in asphyctic pups (Figure 2). More than a two-day-delay was observed in the ear twitch and crossed extensor reflexes due to the hypoxic exposure. Enriched environment itself led to earlier development of crossed extensor reflex and auditory startle reaction. Furthermore, environmental enrichment could significantly diminish the developmental delay in the crossed extensor reflex (Figure 2A). Limb placing testing showed that asphyxia caused a significant delay in the appearance of both forelimb and hindlimb placing (Figure 2B,C). Environmental enrichment led to an earlier appearance of forelimb and hindlimb placing reflexes and could counteract the negative effects of the asphyctic lesion. Similar results were obtained in the grasp reflexes. Both forelimb and hindlimb grasp reflexes appeared significantly later in pups undergoing asphyxia, while environmental enrichment could decrease this delay (Figure 2B,C). This ameliorating effect by enrichment was significant in case of forelimb grasp. Marked differences were observed in the air righting reflex: a more than 1.5 day delay was caused by asphyxia, which was not significantly decreased by enriched environment (Figure 2D).

**Figure 3:**
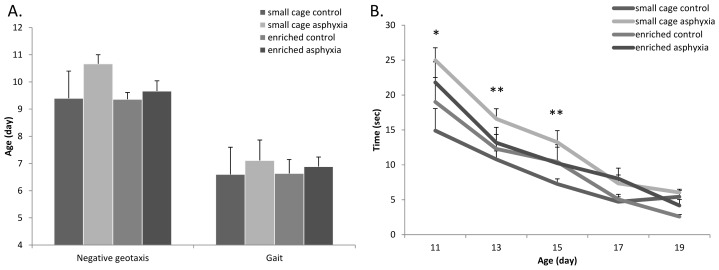
(**A**) Negative geotaxis and gait appearance; (**B**) Gait test performance (* *p* < 0.05, ** *p* < 0.01 small cage control *vs.* small cage asphyxia).

There were no marked differences in the appearance of negative geotaxis and gait reflexes between the different groups (Figure 3A). However, the tendency of a delay in the small cage control asphyctic group could be observed. It took asphyctic pups longer to move out of the circle in the gait test. This time was slightly less in the enrichment group, but results were not significantly different (Figure 3B).

### 2.4. Motor Coordination

**Figure 4:**
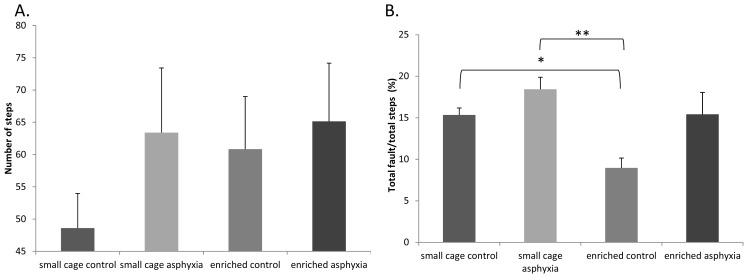
Motor coordination tests: Grid walking and footfault test. (**A**) Number of total steps; and (**B**) Number of total faults (* *p* < 0.05, ** *p* < 0.01).

Among the motor coordination tests, one of the most reliable indicators in our previous studies has been the grid walking/foot fault test [25,26,40]. On counting the number of steps in postnatal week 4, we found that the small cage control rats took fewer steps than all other groups (Figure 4A). Differences, however, were not significant. In the foot-fault test, as expected, asphyctic pups made significantly more mistakes on the elevated grid. Enriched control rats made fewer mistakes than small cage control pups, and a similar tendency could be observed between small cage asphyctic and enriched asphyctic groups (Figure 4B).

### 2.5. Discussion

Our present results show that enriched environment is able to decrease the delay in neurobehavioral development induced by perinatal asphyxia. We found that perinatal asphyxia led to a marked delay in the somatic and reflex development as well as in the maturation of motor coordination. These observations are in accordance with our previous study with perinatal asphyxia [28]. We have also described earlier that enriched environment is able to counteract the deleterious effects of excitotoxic injury induced by neonatal monosodium glutamate treatment [39]. Thus, our present study is a confirmation of the beneficial effects of enriched environment in neonatal neurobehavioral development and our results also provide an additional protective strategy in perinatal asphyxia.

Perinatal asphyxia has been shown to induce cognitive, locomotor and other behavioral deficits [30]. In the background, several biochemical and morphological alterations have been described. Among others, changes in neurotransmitter levels and metabolic parameters have been found in the hippocampus and cerebral cortex [41–43]. Delayed cell death, fiber sprouting and changes in postsynaptic densities have also been found in the hippocampus [29,30,44]. Recent results indicate that asphyxia can trigger cell proliferation, gliogenesis in particular [45]. Changes in protein ubiquitination in postsynaptic densities have also been reported [46]. Studies also indicate that there is an acute proinflammatory response in the brain following asphyxia [47,48]. Increased apoptotic cell death and neuronal loss have been described in the striatum and substantia nigra [49,50]. Changes not only in the brain, but in sensory organs and even in the periphery have been described [47,51]. Earlier we have found that perinatal asphyxia leads to severe degeneration of the rat retina [28].

In the present study we confirmed previous findings that perinatal asphyxia leads to severely delayed neurobehavioral development. Among the physical parameters we observed reduced weight gain and delayed appearance of the physical maturation signs in pups exposed to asphyxia. However, rats kept in enriched cages did not show a difference in their weight from the controls after the first week and the delay in the eye opening and incisor eruption was also decreased. Delays in the appearance of reflexes and also motor coordination maturation could be effectively ameliorated.

The beneficial effects of enriched environment have been known since the first description of Donald Hebb [35], who took experimental rats home as pets and observed that these rats performed significantly better in cognitive tests after taking them back to the laboratory. Since then, numerous studies have confirmed these initial findings and have aimed to explore the mechanisms in the background. Enriched environment has been shown to influence the development of the nervous system including that of the visual system [52,53]. Behavioral parameters that have been described to be altered under enriched conditions include less stereotypic repetitive movements [54], decreased age-related impairments in learning [55], reduced depressive-like symptoms [56], attenuated response to psychostimulants [57] and effects on risk-taking behavior [58]. Enriched environment not only influences normal development and decreases pathological behavioral patterns but it also protects against various injuries affecting the nervous system. These include ischemic, toxic and traumatic injuries [59,60]. More precisely, environmental enrichment reduces both functional deficits and morphological lesions in 6-OHDA-induced lesion [61], cortical impact-induced traumatic brain injury [62] or neonatal hypoxic-ischemic injury [63]. Also, neonatal enrichment can reverse the effects of isolation rearing [64]. Amelioration of sensory functions has also been described upon exposure to enriched environment, including visual performance and protection in retinal degeneration [37,65]. Recently we have shown, for the first time, that environmental enrichment has a protective effect in neonatal excitotoxic lesion of the retina [37,66], and in adult ischemic retinal lesion [38]. Regarding neonatal injuries, several positive effects of environmental enrichment have been shown. Our earlier studies have described that enriched environment is able to reverse some of the deleterious effects of glutamate toxicity on neurobehavioral development and protect the retina against excitotoxicity-induced degeneration [28,39]. For a neonatal frontal injury, an enriched environment has been demonstrated to increase cortical thickness along with amelioration in cognitive performance [67]. Pups kept in an enriched environment have been shown to overcome some behavioral problems after perinatal alcohol exposure [68] or in post-traumatic stress disorder [69]. Some studies have shown beneficial effects of enriched environment in neonatal hypoxic injuries. For example, in a hypoxic-ischemic neonatal injury, authors have found that environmental enrichment has reversed the deficits in spatial reference and working memory impairments, but had no effect on hippocampal or cortical morphology [70]. Another study has found that rats exposed to neonatal anoxia had drastically altered social and self-control behaviors, which was reversed by enrichment [71].

The exact molecular mechanism of this protective effect is not fully known. Mechanisms that lead to cell death after asphyxia include disturbance of mitochondrial energy mechanisms, increased activation of poly-ADP ribose polymerase, opening of mitochondrial membrane permeability transition pore and inactivation of key, rate-limiting metabolic enzymes, e.g., the pyruvate dehydrogenase complex [72]. However, the activation of several beneficial molecular pathways has been described in animals exposed to environmental enrichment. Enrichment influences the activation of mitochondrial and non-mitochondrial apoptotic pathways, involving Bax and Bad proteins, caspases, and mitogen activated protein kinases (e.g., p38 MAPK) [72,73]. In addition, changes in nitric oxide synthase expression and decrease in oxidative stress markers were described in a recent study [74]. Furthermore, stimulated neurogenesis, increase of dendritic spines and elevated expression of neurotrophic factors like brain-derived neurotrophic factor, insulin-like growth factor and nerve growth factor [52,75–77] have been described. These growth factors induce synaptogenesis, and motor protein changes necessary for plasticity after injuries in enriched conditions [78]. Serotonin receptor density was described as normalized in anoxic rats after environmental enrichment [71], as well as noradrenalin-stimulated calcium increment in some, but not all, areas of the hippocampus [79]. Actions of environmental enrichment on hippocampal glucocorticoid receptors [77] and nerve growth factor concentration have also been reported [77]. Even if the protective mechanism is not fully understood at the moment, enriched environment can be a promising strategy in treating asphyctic newborns also in human clinical therapy. Similarly, the neuroprotective effects of tactile stimuli were first described in rats, and are now used in neonatal care, as part of standard handling [80].

## 3. Experimental Section

### 3.1. Experimental Animals

A local Wistar rat colony was used for our experiments. Animal housing, care and application of experimental procedures were in accordance with institutional guidelines under approved protocols (No: BA02/2000-15024/2011, University of Pecs following the European Community Council Directive). Animals of both sexes were cross-fostered immediately after birth, to minimize litter differences. Litter size was 8 ± 1 pups in all groups. Pups stayed with their mothers during the whole examination period and were weaned after 5 weeks of age. All experimental animals were kept in the same room, under the same illumination and other outside environmental conditions (12 h light-dark cycle, food and water *ad libitum*).

### 3.2. Asphyxia

Female Wistar rats were inspected for vaginal sperm plug and then the pregnant animals were observed on gestational day 22. As soon as delivery started, mothers were sacrificed by neck dislocation under anesthesia [2,28,81]. Pups were delivered by caesarian section from the uterine horns and stimulated to breathe. Control cesarean-delivered pups were delivered immediately (*n* = 25), while other pups were delivered following a 15-min asphyctic period (*n* = 21), kept at constant temperature (37 °C). Asphyxia was achieved by leaving the pups in the uterus for 15 min without breathing. Surviving rats were given to surrogate mothers after a survival period of 40 min at 37 °C. Neurobehavioral maturation and development of motor coordination were tested based on earlier descriptions [25,26,82–87]. Only the surviving animals are included in the final evaluation. During the acute post-asphyctic phase, more than 50% of pups died in the asphyxia group, compared to the 10% of the control group. Also, during the observation period, 4 pups died in the asphyxia group, while none in the control group. Gender was evenly distributed in the groups (55% males, 45% females).

### 3.3. Environmental Enrichment

**Figure 5:**
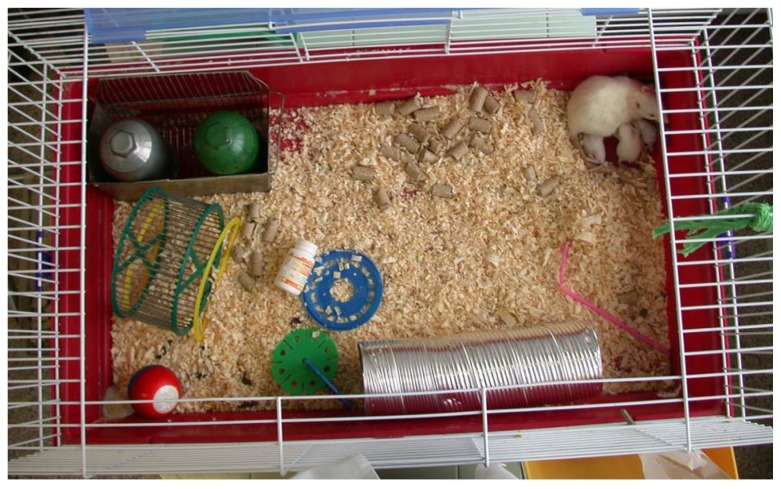
Enriched environment.

Pups were placed in one of the following two cages immediately after birth, similarly to our earlier descriptions [37]. (1) Normal control rats were placed in a regular (control) cage with 43 × 30 × 20 cm^3^ dimensions (*n* = 10 control, *n* = 9 asphyxia). Control rats were only shortly handled, for the duration of the neurobehavioral testing. (2) A second group of pups (*n* = 15 control, *n* = 12 asphyxia) was placed in a large cage, the floor of which was 88 × 50 cm^2^ with 44 cm high walls (88 × 50 × 44 cm^3^) supplemented with a complex environmental enrichment (Figure 5). Rats were continuously exposed to intensive multisensory stimulation. The cage contained different toys, objects, running tunnels and rotating rods with various shapes, materials (wood, plastic, metal), colors and shades. Half of the objects were changed daily, while the other half were left unchanged to avoid a stressful change of the environment.

### 3.4. Examination of Neurobehavioral Development

Examinations of neurobehavioral development were started on the first postnatal day (PND) and were carried out daily between 12 and 15 p.m. until PND 21. Neurobehavioral testing was performed in a blinded fashion, the investigator was not aware of the nature of handling. Bodyweight was measured daily until 3 weeks of age, then twice a week until 5 weeks of age. Physical development was followed by inspections of maturation of physical characteristics such as eye opening, incisor eruption and ear unfolding. Pups were also tested for the following neurological signs and reflexes: (1) Negative geotaxis: animals were placed head down on an inclined grid (45°) of 30 cm. The hindlimbs of the pups were placed in the middle of the grid. The day they began to turn around and climb up the board with their forelimbs reaching the upper rim was noted. In cases where the animal did not turn around and climb up the board within the observed 30 s, the test was considered negative. From the day of the appearance of the negative geotaxis, the time in seconds to reach the upper end of the board was recorded daily; (2) Crossed extensor reflex: the left rear paw was pinched and the animal was observed for the extension of the right leg. The day of disappearance of the crossed extensor reflex in its pure form, when it was replaced by a more complex behavioral response, was noted; (3) Sensory reflexes: the ear and the eyelid were gently touched with a cotton swab and the first day of the ear twitch reflex and the contraction of the eyelid were recorded; (4) Limb placing: the back of the forepaw and hindpaw was touched with the edge of the bench while the animal suspended, and the first day of lifting and placing the paws on the table was noted; (5) Limb grasp: the fore- and hindlimbs were touched with a thin rod, the first day of grasping onto the rod was recorded; (6) Gait: the animals were placed in the center of a white paper with a circle of 13 cm in diameter, the day the pup began to move off the circle with both forelimbs was recorded. In cases the animal did not leave the circle for 30 s, the test was considered to be negative. From the day of the appearance, the time in seconds to move off the circle was recorded daily; (7) Auditory startle: the first day of the startle response (body shaking) to a clapping sound was observed; (8) Air righting: subjects were dropped head down onto a bed of shavings from a height of 50 cm, and the day of first landing on four feet was recorded.

### 3.5. Motor Coordination Tests

Rat pups were tested for motor coordination twice on postnatal week 4. Grid-walking and footfault test: rats were placed on a stainless steel grid floor (20 × 40 cm^2^ with a mesh size of 4 cm^2^) elevated 1 m above the floor. For a 1-min observation period, the total number of steps was counted (calculated by total right and left forelimb steps). The number of footfault, when the animals misplaced a forelimb or hindlimb that it fell through the grid, was also recorded during a 1-min period (mistakes of all four limbs were counted separately during the examination).

### 3.6. Statistical Analysis

Data are expressed as mean ± standard error of the mean (SEM). Statistical analysis was performed using two-way analysis of variance (ANOVA) followed by Bonferroni-Dunn’s posthoc analysis. Results were considered significant when *p* < 0.05. Results are represented with * in case of significance level 0.01 < *p* < 0.05; ** when 0.001 < *p* < 0.01 and *** when *p* < 0.001.

## 4. Conclusions

In conclusion, in the present study we showed that some of the components of the neurobehavioral delay induced by perinatal asphyxia could be overcome by environmental enrichment, which thus is a promising strategy in early neonatal injuries.
